# Advances, Applications, and Emerging Opportunities in Electrostatic Hydrogels

**DOI:** 10.1021/acs.langmuir.3c02255

**Published:** 2023-11-17

**Authors:** Holly Senebandith, Defu Li, Samanvaya Srivastava

**Affiliations:** † Department of Chemistry and Biochemistry, University of California, Los Angeles, Los Angeles, California 90095, United States; ‡ Department of Chemical and Biomolecular Engineering, University of California, Los Angeles, Los Angeles, California 90095, United States; § California NanoSystems Institute, University of California, Los Angeles, Los Angeles, California 90095, United States; ∥ Institute for Carbon Management, University of California, Los Angeles, Los Angeles, California 90095, United States

## Abstract

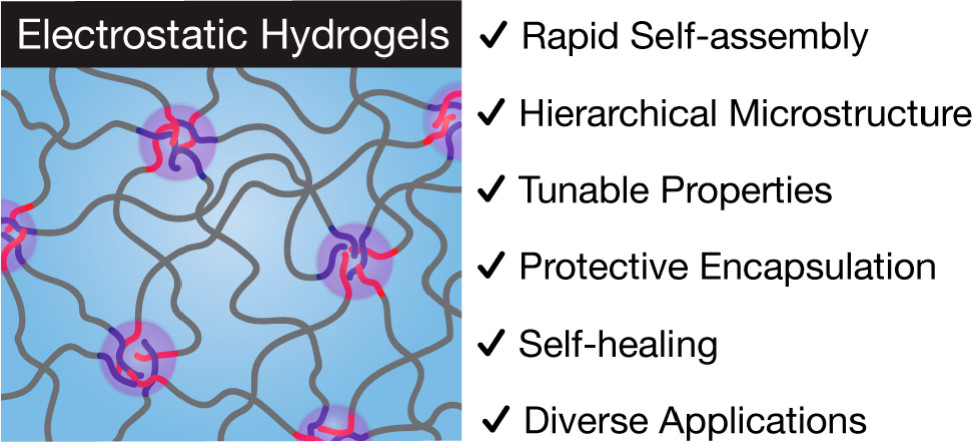

Polyelectrolyte complex (PEC) hydrogels, which self-assemble via complexation of oppositely charged block polymers, have recently risen to prominence owing to their unique characteristics such as hierarchical microstructure, tunable bulk properties, and the ability to precisely assimilate charged cargos (i.e., proteins and nucleic acids). Significant foundational research has delineated the structure–property relationship of PEC hydrogels for use in a wide range of applications. In this Perspective, we summarize key findings on the microstructure and bulk properties of PEC hydrogels and discuss how intrinsic and extrinsic factors can be tuned to create specifically tailored PEC hydrogels with desired properties. We highlight successful applications of PEC hydrogels while offering insight into strategies to overcome their shortcomings and elaborate on emerging opportunities in the field of electrostatic self-assemblies.

## Introduction

Self-assembled hydrogels encompass a broad diversity of materials in which polymers are linked together into three-dimensional (3D) networks via noncovalent (and typically reversible) interactions, such as electrostatic interactions, hydrophobic interactions, van der Waals forces, π–π stacking, hydrogen bonding, metal coordination, and host–guest interactions.^1,2^ The reversible interactions supporting the physical networks enable excellent self-healing and recovery behavior^3−10^ as well as responsiveness to stimuli in such hydrogels.^3,9−17^ These features make them highly desirable in biomedical and consumer product applications.^1,18^

**Figure 1:**
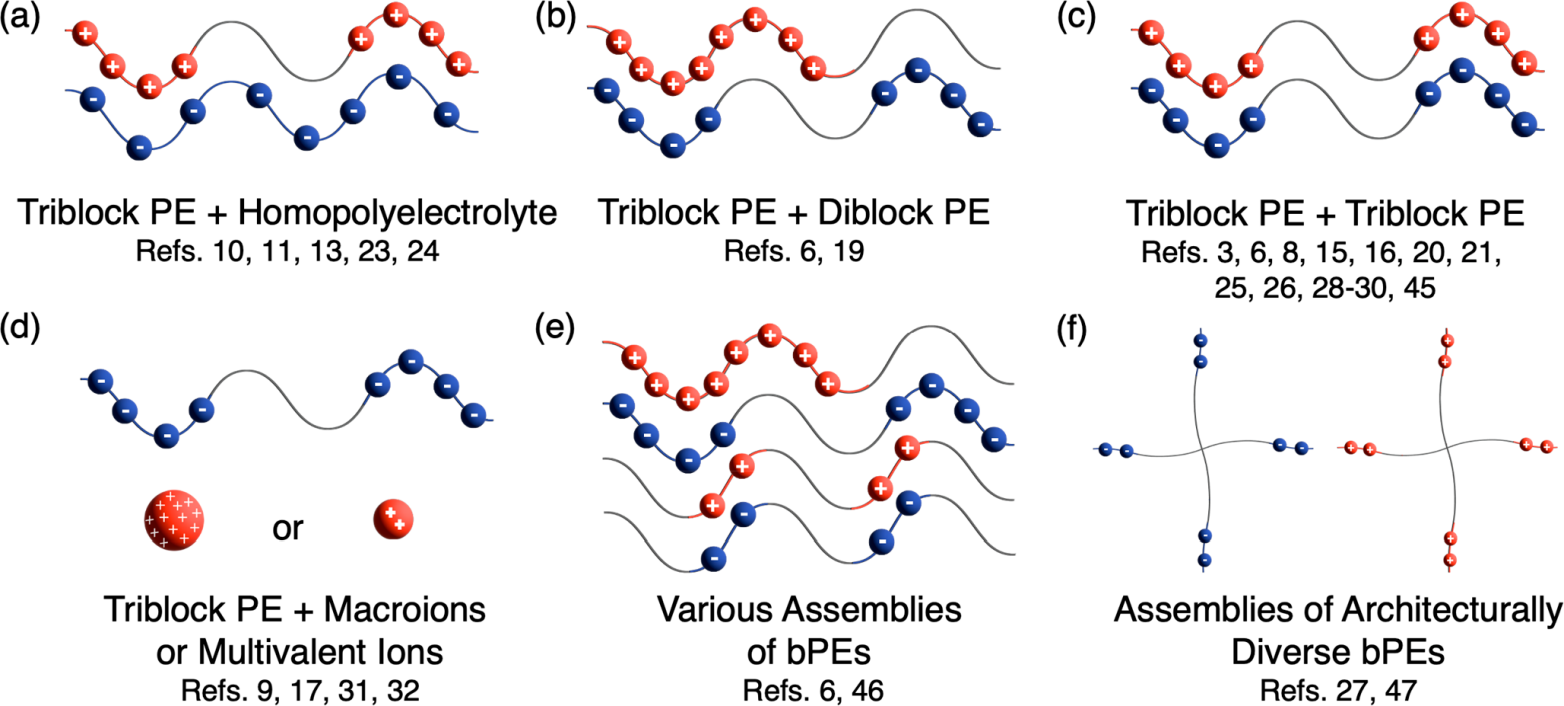
General pathways for making polyelectrolyte complex (PEC) hydrogels. Abbreviations: PE, polyelectrolyte; bPEs, block polyelectrolytes.

These benefits proliferate in hydrogels in which electrostatic interactions drive self-assembly because the long-range electrostatic interactions enable faster self-assembly and greater tunability.^15,19^ Broadly speaking, electrostatic hydrogels are 3D water-laden polymer networks that are physically connected by reversible ionic interactions. Numerous approaches have been developed for the preparation of electrostatic hydrogels, from simple ionically cross-linked polyelectrolytes^17^ to more intricate assemblies of block polyelectrolytes,^3,6,8,10,12,13,15,16,19−30^ macroions,^31^ multivalent coordinating ions/metals,^32^ and charged surfactants (Figure 1).^14^

A classic example of electrostatic hydrogels is the self-assembly of alginate biopolymers and divalent calcium ions.^33^ Alginate is a naturally derived anionic biopolymer with inherent biocompatibility and biodegradability, low toxicity, relatively low extraction cost, and high abundance. Advantages, like their similarity to extracellular matrix (ECM), have made alginate hydrogels attractive in tissue regeneration and repair applications.^33^ However, such biopolymer-based hydrogels (other examples have employed chitosan, pectin, etc.) suffer from the inherent variability of properties associated with biopolymers, such as variability between batches, the possibility of pathogenic contamination, and a significant lack of tunability. Another class of electrostatic hydrogels is polyampholyte hydrogels, formed by the random copolymerization of cationic and anionic monomers.^7^ These hydrogels, composed of polymer networks with both covalent and electrostatic linkages, are usually very tough and self-healing.^7^ However, while there are benefits to using such electrostatic hydrogels, a lack of hierarchical microstructure limits their physicochemical and mechanical tunability.

Microstructure in self-assembled materials has been explored in amphiphilic block copolymers for decades.^34−36^ Melt phase experiments have demonstrated a rich diversity of microstructures in self-assembled amphiphilic block copolymers, such as disordered (DIS) and body-centered cubic (BCC) spheres, hexagonally closely packed cylinders (HCP), lamellae (LAM), and gyroid (GYR) phases, among others.^34−36^ Similar microstructural diversity has also been noted in solution phase assemblies.^37−40^ For example, Choi, Bates, and Lodge^39^ investigated the microstructural evolution of a poly(styrene-*b*-ethylene-*alt*-propylene) (PS-PEP) diblock copolymer solution in squalane, a highly selective solvent for the PEP block. Facile modulation of the self-assembled microstructure was demonstrated by tuning the polymer and solvent concentrations with readily accessible DIS, BCC, HCP, and LAM morphologies. The importance of this observation cannot be understated because these microstructures affect the physicochemical (i.e., viscosity, solubility, melting point, glass transition temperature, domain spacing, pore size, and swelling), optoelectronic (i.e., ionic conductivity, absorption, opacity, and photoluminescence), and mechanical properties (i.e., shear and tensile strengths) of these materials. Therefore, controlling the microstructure is vital for creating materials with desired properties.

These outstanding block copolymer studies have inspired attempts to create self-assembled electrostatic hydrogels with hierarchical microstructures. By relying on the complexation of block polyelectrolytes comprising neutral and charged blocks, a new class of multiresponsive materials, named polyelectrolyte complex (PEC) hydrogels or polyion complex (PIC) hydrogels, was introduced by Cohen Stuart and co-workers^13^ and Tirrell, Hawker, and co-workers^12^ in 2010 and 2013, respectively. They also have been termed complex coacervate core hydrogels (C3Gs).^16^ These hydrogels form via complexation of oppositely charged blocks (or polymers) and possess hierarchical microstructures and unique attributes that position them as an appealing materials platform for use in many applications.^41^

In this Perspective, we discuss the recent advances in electrostatic hydrogels, with an emphasis on PEC hydrogels. In the following sections, we first discuss the research on electrostatic hydrogels in recent years with a focus on hydrogel structure and bulk properties; we also describe their current limitations and ongoing efforts to address these. Subsequently, we discuss the current and potential uses of electrostatic hydrogels and conclude with our anticipation of the next generation of electrostatic hydrogel research.

## Recent Advances in Electrostatically Assembled PEC Hydrogels

Electrostatic PEC hydrogels utilize block polyelectrolytes (bPEs) that self-assemble into physically linked networks, with nanoscale polyelectrolyte complex domains serving as the netpoints (Figure 1). PEC hydrogels boast attributes that are significant for numerous practical applications, such as hierarchical microstructure,^12,30^ swift self-assembly, and the ability to protect charged cargos (e.g., proteins, enzymes, and nucleic acids)^42,43^ in aqueous PEC domains,^3,22,28^ along with other distinguishing properties such as responsiveness to stimuli, self-healing characteristics,^3,6,9,10^ and predictable physicochemical and mechanical properties.^44^

Generally, PEC hydrogels are formed through similar pathways and, consequently, share commonalities in their structure and properties. Typical PEC hydrogels comprise a bPE as at least one of the two (or more) oppositely charged components. As such, the simplest way to create a PEC hydrogel is by mixing a triblock polyelectrolyte with an oppositely charged homopolyelectrolyte (Figure 1a),^10,11,13,23,24^ diblock polyelectrolyte (Figure 1b),^6,19^ or triblock polyelectrolyte (Figure 1c).^3,6,8,15,16,20,21,25,26,28−30,45^ It is also possible to create a PEC hydrogel by mixing a triblock polyelectrolyte with oppositely charged macroions^9,31^ or multivalent metal ions (Figure 1d).^17,32^ Hydrogels with assemblies of oppositely charged bPEs [i.e., diblock, triblock, and pentablock (Figure 1e)] have also been demonstrated.^6,46^ Finally, in addition to linear polyelectrolytes, PEC hydrogels have also been created from oppositely charged bPEs possessing nonlinear architectures [e.g., three- and four-arm bPEs (Figure 1f)].^27,47^ It should be noted that these combinations are not an all-encompassing description of PEC hydrogel fabrication pathways; other combinations may exist.

In addition to the choice of components, intrinsic parameters such as the length of the charged and neutral polymer blocks, the polymer concentration, and the nature of the charged groups can be manipulated to achieve precise control over the design and properties of PEC hydrogels. Variation of the intrinsic parameters affects the network microstructure, including the PEC domain morphology and the distance between domains (i.e., mesh size), which in turn affects the moduli, salt resistance, and other physical properties of the hydrogels.^9,30^ PEC hydrogels also benefit from another level of tunability provided by the variation of externally controlled parameters. For example, upon the addition of salt (e.g., NaCl), the electrostatic interactions can be screened, thus affecting the moduli and microstructure of the hydrogel.^3,9,12,13,16,22,23,25,47^ For PEC hydrogels with functional groups that are weakly ionized, pH is another external parameter that can be used to tune their microstructure and properties.^13,47^ Multiple levels of tunability distinguish PEC hydrogels as a clear choice for precisely designed materials.

### Hierarchical Microstructure of PEC Hydrogels

**Figure 2:**
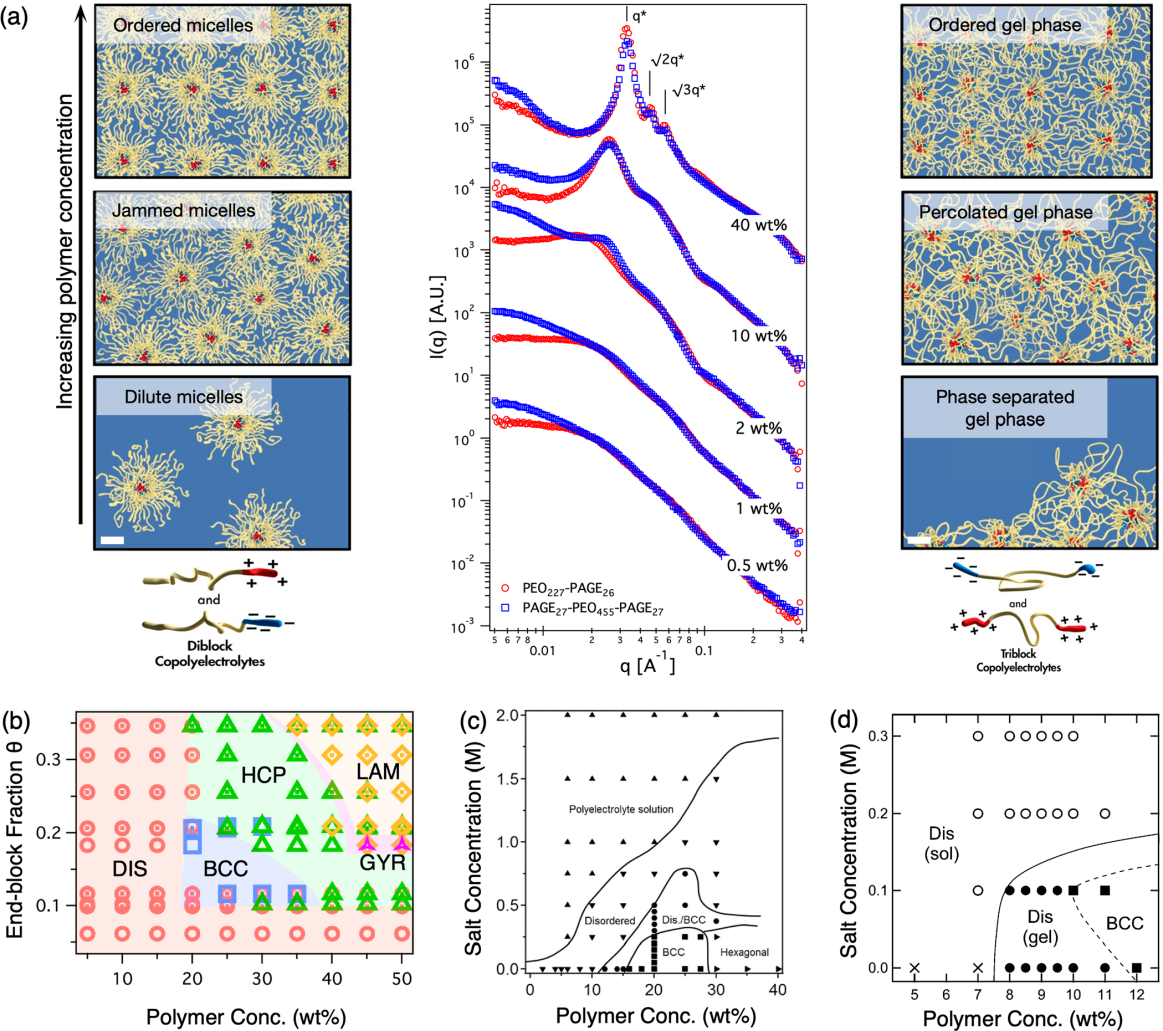
Hierarchical PEC hydrogel microstructures. (a) Progression of the self-assembly of oppositely charged diblock polyelectrolytes (left) and triblock polyelectrolytes (right) in the structural progression of polyelectrolyte complex (PEC) hydrogels. Diblock bPEs form star-like micelles, while triblock bPEs form phase-separated gels. As the polymer concentration increases, diblock micelles become jammed, while triblock micelles begin to bridge into a percolated gel. Eventually, both diblock and triblock PE self-assemblies form ordered microstructures and undergo several order–order transitions. Scattering spectra of diblock (red) and triblock (blue) PEC self-assemblies with increasing polymer concentrations as a function of wave vector *q* (center) reveal their microstructural evolution. Scale bars are 15 nm. Adapted from ref (29). Licensed under CC BY 4.0. (b) Experimental morphology map of PEC hydrogels as a function of polymer composition and concentration. The symbols denote the following: red circles for disordered (DIS) spheres, blue squares for body-centered cubic (BCC) spheres, green triangles for hexagonally closely packed (HCP) cylinders, orange diamonds for parallelly stacked lamellae (LAM), and pink three-pointed stars for the gyroid (GYR). Adapted with permission from ref (30). Copyright 2020 American Chemical Society. (c and d) Experimental morphology maps of PEC hydrogels as a function of polymer and salt concentrations. In panel d, the disorder–order transition appears at a lower polymer concentration of 12 wt% due to the longer charged block lengths of bPEs. Panel c adapted with permission from ref (12). Copyright 2013 American Chemical Society. Panel d adapted with permission from ref (3). Copyright 2020 American Chemical Society.

The electrostatic self-assembly in PEC hydrogels is hypothesized to proceed in a manner analogous to that of PEC micelles. Let us consider the representative example of aqueous self-assembly of oppositely charged ABA triblock polyelectrolytes. At very low concentrations, the polymers are expected to self-assemble into flower-like micelles, consisting of a polyelectrolyte complex (or complex coacervate) core comprising the oppositely charged A blocks surrounded by a corona comprising loops of neutral B blocks.^9,11,29^ A similar assembly can be envisioned in aqueous mixtures of oppositely charged AB diblock polyelectrolytes, as well, resulting in star-like micelles with coronae consisting of dangling B blocks (Figure 2a, left).^19,29^ Representative micelle core radii of ∼8 nm were reported (Figure 2a, left).^29^ As the bPE concentration increases, the intermicellar distances decrease, resulting in the overlap of their coronae. In the case of ABA triblock polyelectrolytes, a major fraction of the looping B blocks also begin forming bridges between the micelles, resulting in networks with PEC domain netpoints (Figure 2a, right).^3,8,12,13,16,23,24,28,29^

At higher bPE concentrations, strengthening correlations among the PEC domains, their ordering, and subsequent morphological transitions are noted for both di- and triblock polyelectrolyte assemblies, which is evident from small-angle scattering spectra (Figure 2a, center).^3,12,15,19,20,22,25,30^ The systematic progression of domain microstructure, evolving from disordered spheres (DIS) to spherical domains arranged in a BCC lattice, to HCP cylinders, and subsequently to parallelly stacked lamellar domains, along with coexisting domain morphologies, has been reported in PEC hydrogels (Figure 2b).^12,30^ This progression in PEC hydrogels is analogous to the structural evolution in amphiphilic block copolymers,^35,39^ but it is imperative to note that electrostatic interactions drive the self-assembly in the former as opposed to solvophobic interactions and/or chemical incompatibility among the blocks of the polymers in the latter. Another notable difference between the electrostatic assembly of oppositely charged ABA triblock polyelectrolytes and the solvophobic assembly of ABA triblock polymers is the nearly 10-fold difference between the polymer concentrations at which bridging between the flower-like micelles results in the formation of networks.^29^ In the latter, network formation and percolation occur at similar polymer concentrations, resulting in the formation of gels. In the former, bridging occurs at significantly lower concentrations, resulting in networks that cannot percolate the system and, therefore, form phase-separated gels (Figure 2a, right).^29^ This disparity is revealed from the differences among the scattering spectra from the diblock and triblock assemblies at low bPE concentrations of ≤2 wt%, wherein the triblock PE self-assemblies exhibit a subtle correlation peak in the vicinity of 0.02–0.03 Å^–1^, while the diblock PE self-assemblies exhibit characteristic form factor scattering spectra (Figure 2a, center). These findings were also supported by molecular dynamics simulations.^29^ We expect these microstructural features, including strengthening correlation among PEC domains with increasing polymer concentration, the emergence of ordered microstructure, and morphological transitions, to be representative of PEC hydrogels and return to discussing generic microstructural trends in PEC hydrogels for the remainder of this section.

**Figure 3:**
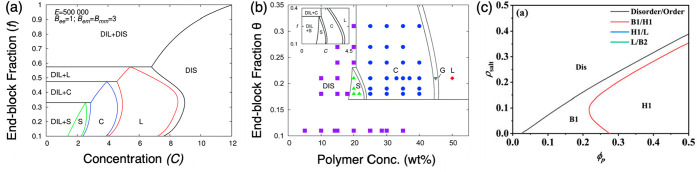
Theoretical phase diagrams. (a and b) Theoretical and experimental morphology maps, with the latter obtained from an SCFT-EF model, as a function of polymer concentration and end block fraction. The inset of panel b depicts a theoretical morphology map with a smaller parameter range. Abbreviations: DIL, dilute; S, BCC or face-centered cubic spheres; C, HCP cylinders; G, gyroid; L, lamella. Reproduced (adapted) with permission from ref (20). Royal Society of Chemistry; permission conveyed through Copyright Clearance Center, Inc. (c) Theoretical morphology map as a function of salt concentration (ρ_salt_) and polymer concentration (ϕ_p_) with a ratio of charged blocks to uncharged blocks of 0.3, agreeing with experimental data from ref (19). Abbreviations: Dis, disordered; B1, BCC; H1, HCP. H1/L (blue line) and L/B2 (green line) are not observed in the phase diagram. Reprinted with permission from ref (48). AIP Publishing.

To maintain the ease of handling and working with limited amounts of polymers, many studies have remained restricted to polymer concentrations of <20 wt%, precluding observations of ordered microstructures.^3,6,13,14,16,21−24,27^ Scattering studies on hydrogels with polymer concentrations of ∼10–20 wt% suggest a strong spatial correlation among PEC domains (Figure 2a, center),^13,16,23,24,28,29,45^ yet structural ordering is normally not observed until higher polymer concentrations are reached. Increasing the polymer concentration beyond 20 wt% reveals a disorder–order transition from DIS to BCC, usually between ∼14 and ∼25 wt%^12,15,19,20,30^ followed by an order–order transition from BCC to HCP at >25 wt% (Figures 2c,d and 3a–c).^12,19,20,30^ At higher polymer concentrations (35–50 wt%), HCP to gyroid (GYR) to LAM order–order transitions have also been observed (Figures 2c and 3b).^20,30^

Though the structural progression has a general pattern, it should be noted that microstructural transition points depend on intrinsic and extrinsic system parameters. Intrinsically, the lengths of the charged and the neutral blocks, the charge density of the charged blocks, the polymer concentration, and the nature of the ionizable groups affect the hydrogel microstructure. For instance, BCC ordering at a polymer concentration of 12 wt% was noted by Kim et al.^3^ in triblock polyelectrolyte assemblies with long charged block lengths [charged block degree of polymerization (DP) of 78 (Figure 2d)]. Hydrogel microstructure can also be modulated by varying extrinsic factors like salt, pH, temperature, and crowding agents.^11,20,26,30^ For instance, Krogstad et al.^12,19^ and Kim et al.^3^ have presented the microstructural evolution of PEC hydrogels as a function of polymer concentration (intrinsic factor) and salt concentration (extrinsic factor), highlighting how they affect hydrogel microstructure inversely and, therefore, can be employed in tandem to modulate the microstructure.

Theoretical models predicting PEC hydrogel microstructure support the experimental findings. Audus et al.^20^ reported the first theoretical phase diagram (Figure 3a) using self-consistent field theory with embedded fluctuations to describe the microstructure of PEC hydrogels comprising ABA bPEs, noting that the embedded fluctuations were necessary to incorporate the influence of long-range electrostatic correlations. While a qualitative similarity between the theoretical (Figure 3b, inset) and experimental phase diagram was noted (Figure 3b), their model was unable to account for high-charge density polymers. Jiang et al.^48^ resolved this with their theoretical model by embedding ion pairing explicitly in their SCFT model, leading to a phase diagram (Figure 3c) that qualitatively agreed with previous experimental results.^12,15,19,29^ In particular, the authors stressed that their theoretical calculations for a system with an end block fraction of <0.3 revealed only B1 (BCC) and H1 (HCP) ordered phases (Figure 3c), similar to the phase diagram reported by Krogstad et al.,^19^ with the transition from BCC to HCP for no-salt systems occurring at a polymer concentration of ∼30 wt%.

### Tunable Viscoelastic Properties of PEC Hydrogels

The viscoelastic properties of PEC hydrogels can also be modulated by modifying intrinsic parameters like the assembly pathway, polymer concentration, length of the charged and neutral blocks, nature of the ionizable groups, and charge density of the charged blocks, as well as extrinsic parameters like salt concentration, pH, and crowding agents that can alter the connectivity of the PEC network. Depending on these parameters, different ranges of moduli are noted for different PEC hydrogel systems. For example, triblock and homopolymer (Figure 1a), triblock and triblock (Figure 1c), and PEC hydrogels with various assemblies of bPEs (Figure 1b,e) report moduli from <1 to >10 000 Pa.^6,10,11,19,23,30,49^ Systems of triblock bPEs and macroions or multivalent ions (Figure 1d) have reported moduli ranging from 1000 to 30 000 Pa^9^ or <3000 Pa,^17^ respectively. Lastly, PEC hydrogels made from nonlinear bPEs (Figure 1f) have reported moduli of <4000 Pa.^27^

**Figure 4:**
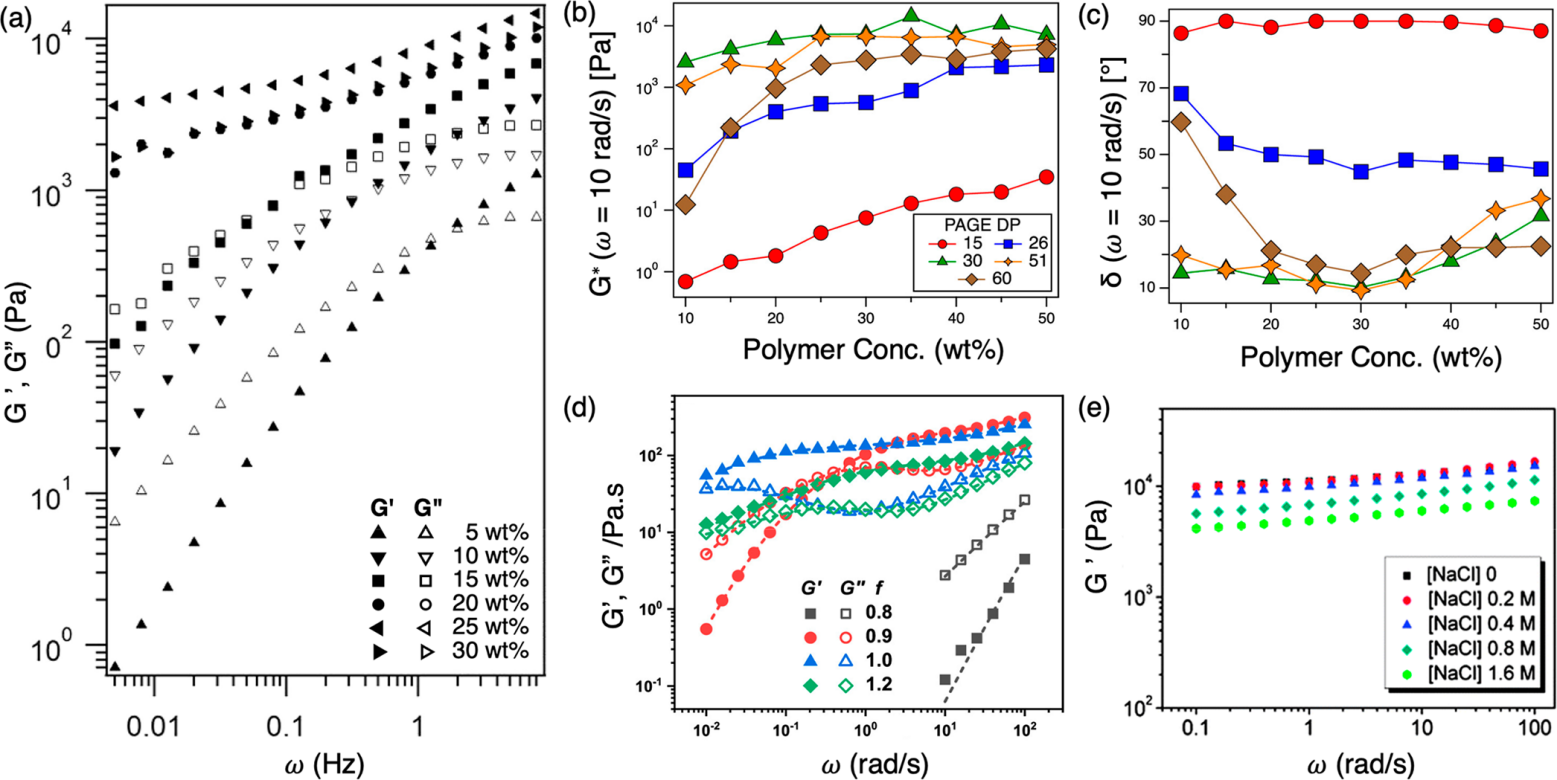
Tunable viscoelastic properties of PEC hydrogels. (a) Storage (*G*′) and loss (*G*″) moduli as a function of angular frequency (ω) for PEC hydrogels with an increasing polymer concentration. Adapted with permission from ref (12). Copyright 2013 American Chemical Society. (b and c) Complex modulus (G*) and phase angle (δ) as a function of polymer concentration for PEC hydrogels with increasing charged block lengths. Adapted with permission from ref (30). Copyright 2020 American Chemical Society. (d) *G*′ and *G*″ as a function of ω for PEC hydrogels with varying charge ratios (*f*). Adapted with permission from ref (14). Copyright 2021 American Chemical Society. (e) *G*′ as a function of ω of PEC hydrogels with increasing salt (NaCl) concentrations. Reproduced (adapted) with permission from ref (9). Royal Society of Chemistry; permission conveyed through Copyright Clearance Center, Inc.

The large range of moduli, which can be achieved through varying intrinsic and extrinsic factors, is unique to PEC hydrogels. For instance, the polymer concentration can be tuned to influence the hydrogel viscoelasticity. An increasing polymer concentration results in a higher number density of PEC domains,^3,30^ leading to denser networks accompanied by an increase in hydrogel viscosity and moduli (Figure 4a).^4,6,12,13,15,21,27,30,32,49^ Similarly, increasing the charged block length is expected to slow chain relaxation by providing a stronger association between the charged blocks, thus contributing to higher moduli and viscosities.^3,8,27^ Evidently, Choi and co-workers^3^ showed that increasing the charged block length resulted in longer relaxation times due to the thermodynamic energy barrier that polymer chains encounter at the PEC–water interface. At a polymer concentration of 9 wt%, this manifested in increasing moduli as the charged block length increased. However, it should be noted that increasing the charged block length while keeping the polymer concentration constant can result in nonmonotonic trends in moduli (Figure 4b), which has been attributed to the competing effects of increasing chain aggregation numbers in the PEC domains that result in larger PEC domains^30^ with slower chain relaxation,^3^ coupled with a continually decreasing network density.

At high polymer concentrations, the moduli eventually plateau and fluctuate rather counterintuitively (Figure 4a–c). However, upon juxtaposition against the hydrogel microstructure, it becomes evident that these trends in the moduli are inherently tied to the hydrogel microstructure. For example, in a triblock PEC hydrogel with roughly 30-mer charged block ends, gels with the BCC morphology (typically noted around 20–25 wt%) exhibited moduli higher than those of gels with HCP morphology [∼30–40 wt% (Figures 2b and 4a,b)].^12,30^ This is also supported by the trends in the phase angle (for Newtonian fluids, δ = 90°, and for elastic solids, δ = 0°); as the polymer concentration increased, the phase angle decreased, followed by an increase due to changes in PEC domain morphology (Figure 4c).

A PEC hydrogel network can also be strengthened intrinsically by utilizing strong ionizable groups in the charged blocks (e.g., converting ammonium into guanidinium). This increase in moduli can be attributed to stronger complexation among polyelectrolytes with a higher fraction of ionized groups.^15,16^ The strong ionizable groups can also contribute to stronger hydrophobic interactions and hydrogen bonding. As such, it is also possible to precisely modulate the viscoelastic response of PEC hydrogels by combining weak and strong bPEs, as Choi and co-workers recently demonstrated.^16^ Chen and co-workers^14^ proposed another route for modulating the viscoelastic response of PEC hydrogels by varying the ratio of the oppositely charged bPEs. When there is a charge imbalance, the hydrogel cannot form as many PEC domains as when the charge ratio is 1. Therefore, the highest modulus for a hydrogel system is achieved with a stoichiometric or nearly stoichiometric charge ratio (Figure 4d).^14,24,48^

Substantial research has also delved into utilizing externally controlled factors, such as solution ionic strength, to tune the viscoelasticity of PEC hydrogels.^3,12,13,15^ The addition of monovalent salts enhances the screening of electrostatic interactions, weakening the association between the oppositely charged moieties and decreasing the moduli of PEC hydrogels (Figure 4e).^3,11−13,15^ Similarly, for PEC hydrogels composed of weak ionizable groups, adjusting the pH can also alter the moduli by altering the fraction of ionized groups and the strength of complexation.^13,21^ These examples demonstrate how PEC hydrogel moduli can be tuned to a desirable level by simply adding salt or changing the pH. Having multiple avenues for property tunability through internal and external factors presents exciting opportunities for applications of PEC hydrogels.

### Limitations of PEC Hydrogels and Approaches to Address Them

The practical utility of PEC hydrogels has remained limited owing to a few critical shortcomings, which can be broadly classified as material deficiencies, including a low (<10 kPa) shear strength and a negligible tensile strength, and biomedical deficiencies, such as poor biocompatibility and biodegradability and a nanoscale mesh size (interdomain distance). The primary contributors to these shortcomings are the reversible electrostatic interactions that drive the assembly of PEC hydrogels and the bioincompatibility of typical PEs and bPEs. To address these challenges, careful material selection and precise design, synthesis, and engineering of the PEs and bPEs are required.

The adoption of PEC hydrogels comprising synthetic bPEs in biomedicine faces challenges such as high toxicity, low biocompatibility, and low biodegradability of the bPEs.^44^ For example, polycations are toxic to cells because their cationic moieties can damage cellular membranes by interacting with anionic phospholipids and disrupting the ion charge balance inside and outside the cellular membrane.^46^ However, it has been suggested that in the complexed state, the charged moieties may be shielded and hence not present imminent toxicity to the cell.^21,27^ In the development of the appropriate material selection criteria for the next generation of PEC hydrogels, further studies are required to study polyelectrolyte toxicity and understand the relationship between gel toxicity and polymer chemistry, concentration, composition, molecular weight, and charge density. Similarly, typical mesh sizes (interdomain distances) in PEC hydrogels are <50 nm,^30^ which are significantly smaller than cells (∼1–100 μm)^27^ and restrict cell mobility, growth, and proliferation,^18,46^ limiting the utility of PEC hydrogels as tissue engineering scaffolds. Addressing these issues can be pursued by modulation of the gel degradation time by tuning polymer chemistries, lengths, and architectures.

At the same time, the weak shear strength of PEC hydrogels (typically <10 kPa) emerges from their physically cross-linked nature.^12,23,49^ Attempts to bolster the shear strength of PEC hydrogels and imbue tensile strength to them have drawn inspiration from the significant body of research in which complementary polymer networks have been combined to create interpenetrating polymer network (IPN) hydrogels.^7^ Our recent work has combined PEC networks with covalently cross-linked networks to create PEC/covalent IPN hydrogels. In this way, significant improvements in the shear and tensile strength have been achieved along with modulation of the gel swelling behaviors.^25,26^ Future efforts to further modulate the strength of PEC hydrogels can adapt and expand on our approach or bolster the association among the oppositely charged polymers in the PEC domains by augmenting the electrostatic associations with other intermolecular interactions (hydrogen bonding, π–π stacking, cation−π interactions, etc.) or even covalent bonds.

## Applications of PEC Hydrogels

Relatively few studies have tested PEC hydrogels in biological settings. Cui and co-workers^21^ performed *in vitro* and *in vivo* experiments on the biodegradability and cytocompatibility of polypeptide-based PEC hydrogels and reported satisfactory cell viability and cell proliferation in the hydrogels while noting an acute inflammatory response when the hydrogels were placed subcutaneously in rats. Encouragingly, the inflammation dissipated along with the hydrogel after 4 weeks, leaving behind almost completely restored tissue. Further reduction in the inflammatory response can be pursued by including growth factors in the hydrogels that assist in the wound-healing process.

**Figure 5:**
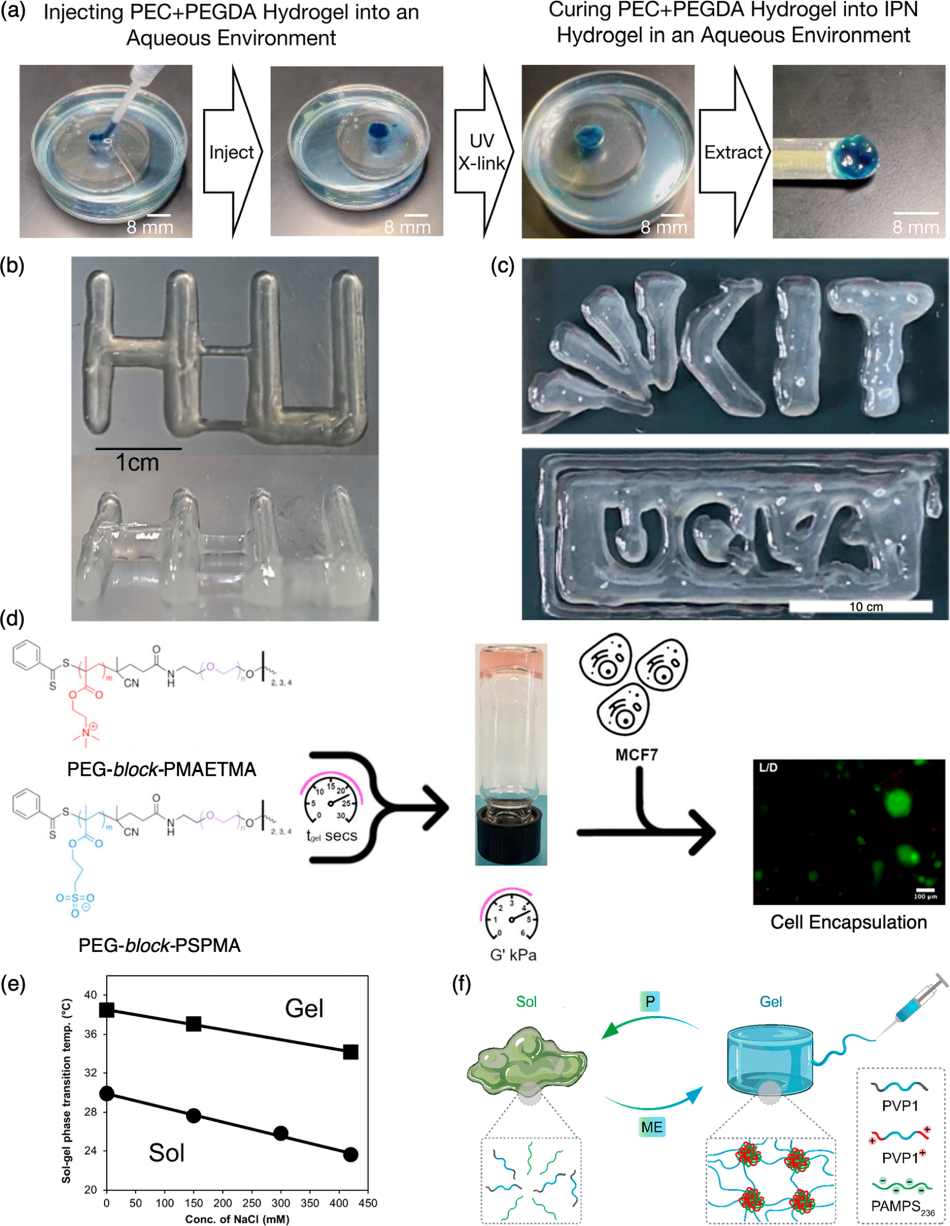
Emerging applications of PEC hydrogels. (a) PEC hydrogels act as protective scaffoldings enabling underwater curing of photo-cross-linkable molecules. Reproduced with permission from ref (26). Institution of Chemical Engineers (IChemE) and the Royal Society of Chemistry; permission conveyed through Copyright Clearance Center, Inc. (b) PEC hydrogels comprising triblock bPEs as inks for extrusion-based 3D printing. Adapted with permission from ref (3). Copyright 2020 American Chemical Society. (c) 3D-printed constructs using 5%/20% GelMA/diblock polyelectrolyte bioinks printed at 37 °C. Adapted from ref (46). Licensed under CC BY 4.0. (d) PEC hydrogels made from star bPE architectures support cell growth. Adapted with permission from ref (27). Copyright 2023 American Chemical Society. (e) Adaptable PEC hydrogels demonstrating the responsiveness to both ionic strength and temperature. Reprinted with permission from ref (11). Copyright 2015 American Chemical Society. (f) Smart PEC hydrogels that can be assembled and disassembled in response to biological signaling molecules. Reproduced with permission from ref (10). Royal Society of Chemistry; permission conveyed through Copyright Clearance Center, Inc.

The swift assembly of PEC hydrogels and their relatively slow swelling rates also enabled the utility of PEC hydrogels as protective scaffoldings in biomedical applications. Our group has also demonstrated underwater curing of photo-cross-linkable precursors enabled by PEC hydrogel scaffoldings, wherein the PEC hydrogels limit the dilution, deactivation, and dissipation of the precursors while still allowing their photo-cross-linking to process in aqueous settings (Figure 5a).^25,26^ These features have been harnessed in a handful of successful studies demonstrating the utility of PEC hydrogels, which portend well for PEC hydrogels as multifunctional biomaterials.

### PEC Hydrogels in 3D (Bio) Printing

Owing to their shear thinning and quick recovery properties, PEC hydrogels make excellent inks in extrusion-based 3D printing. Choi and co-workers,^3^ for example, have demonstrated the 3D printing capabilities of PEC hydrogels (Figure 5b). PEC hydrogels have also been shown to enable the printing of photo-cross-linkable bioinks at physiological temperatures without the need for photo-cross-linking after every deposition step. This allows for the fabrication of defect-free scaffolds in which the dissolution of bPE chains and biodegradation of the bioink allow cell growth and proliferation (Figure 5c).^46,50^

### Cell Scaffolds and Artificial ECMs

The ability to control PEC hydrogel moduli through the precise design of synthetic bPEs has promoted their use as synthetic cell scaffolds for tissue engineering. In a notable study, PEC hydrogels composed of oppositely charged nonlinear bPEs were shown to exhibit tunable stiffness and were tested as scaffolds to support the growth of MCF-7 breast cancer cells. Hydrogels made from the largest three-arm polyelectrolytes (DP of 70) were shown to sustain 96.6% cell viability after 24 h (Figure 5d) and support spheroid MCF-7 tumor formation owing to its appropriate stiffness.^27^ Similarly, Cui and co-workers^21^ incubated mouse fibroblast L929 cells in a polypeptide triblock hydrogel. After incubation for 14 days, they observed increased cell proliferation over conventional two-dimensional culturing systems, demonstrating that PEC hydrogel scaffolds can be beneficial in tissue engineering applications.

### PEC Hydrogels for Drug Delivery

PEC hydrogels have also been shown to serve as effective drug carriers and as scaffolding for the drug carriers, protecting against drug deactivation, unintended biodistribution, and off-target drug effects. Ishii and co-workers^11^ encapsulated an anionic drug into PEC hydrogels and demonstrated responsivity to ionic strength and temperature (Figure 5e) as well as a steady drug release profile. Lee and co-workers^4^ also demonstrated an ionic hydrogel comprising drug-loaded micelles to deliver a cancer drug that delayed tumor growth in mice.

### Smart PEC Hydrogels

Another focus of work in electrostatic hydrogels is the ability to create “smart” materials or materials that are responsive to certain stimuli. Going beyond most studies that report responsivity to salt or pH,^11−13,16,21,23,25,47^ a recent study^10^ has demonstrated the use of activator molecules to stimulate the reversible gelation of a PEC network (Figure 5f) with tunable mechanical strength. Excitingly, these gels exhibited low toxicity and degraded within minutes in response to biochemical signals (i.e., amino acids).

## Opportunities for PEC Hydrogels

Research in recent years has led to great leaps in our understanding of the properties, behavior, and applications of PEC hydrogels and highlighted the remarkable progress that has been accomplished in creating designer PEC hydrogels. However, much remains to be explored.

One of the major ongoing questions is whether our understanding of the composition, chain relaxation processes, and coacervate–water interface translates from bulk PECs to self-assembled PEC hydrogels (and vice versa). As an example, reports employing block and homopolymers of similar chemistry and charged block lengths contrast liquid-like coacervate domains in PEC hydrogels^22,28^ with solid-like bulk precipitates rather than liquid-like droplets.^51^ The mechanisms leading to such disparate behaviors hold the key to controlling the composition of PEC domains in self-assembled gels and the gel processability, self-healing, and swelling properties of PEC hydrogels.

In using PEC hydrogels as therapeutic depots and delivery vehicles, it is still not understood how encapsulating additives within the PEC domains affect the structure, morphology, and behavior of PEC hydrogels. Numerous reports of encapsulation of charged macromolecules within complex coacervates and PEC micelles are expected to inspire and provide the basis for studies exploring additive encapsulation in PEC hydrogels.^42,43^

Lastly, while salt, pH, and temperature have been thoroughly researched,^11−13,16,21,23,25,47^ more imaginative response methods have yet to be realized in PEC hydrogels. For example, photocleavable functional groups could be implemented to create PEC hydrogels that release cargo or degrade in response to long-wavelength visible light. PEC hydrogels could also be engineered to be responsive to changes in mechanical force. Other methods of external responsivity to stimuli that could be incorporated into PEC hydrogels include responsivity to biomolecules (e.g., glucose), electricity, magnetism, reactive oxygen species (ROS), redox, and enzymes.^52^

In summary, electrostatic hydrogels, especially PEC hydrogels, make up an emerging class of soft materials with unique properties that position these as remarkably tunable hydrogels. Research in the field has grown into a very exciting area with the opportunity to create some of the most versatile, rationally designed, and intrinsically valuable materials. With careful design, we anticipate functional PEC hydrogels will be created to cater to a vast range of applications, biomedical or otherwise. With all the possibilities awaiting exploration, we look forward to the, no doubt, revolutionary future of electrostatic self-assemblies.
